# The Rev. Mr. Gale, on the Cow-Pox

**Published:** 1801-02

**Authors:** J. S. Gale

**Affiliations:** East Woodhay, Hants


### *To the Editors of the Medical and Phyfical Journal.*

Gentlemen,

AMONGST the many difcoveries that have been made for the advantage of the public, none furely ever claimed greater attention and gratitude than that of the Cow-pox.

I mean not to enter into any medical difcuffions of this difeafe, but only to ftate a few plain matters of fact, which have paffed through my own hands or under my obfervation.

Having procured the genuine Vaccine matter, I firft inoculated my fervants; one of whom expreffing a wifh to know, by experiment, if it were an effectual preventive of the Small-pox, I permitted him to expofe himfelf to its contagion, which he did, by lying with and inhaling the breath of a boy who was blind, and fuppofed to be in the agonies of death, with this cruel diftemper. From this enterprife my fervant felt no ill effect whatever. The mother of the boy, who was ill with the Small-pox, fhortly afterwards fickened with the fame diftemper, and foon became a deplorable object in it; when her hufband, who dreaded the malady, implored me to inoculate him with the Cow-pox; which I did, and he became an attendant on his wife and fon, in perfect fecurity: But be it underftood, that till he had been inoculated for the Vaccine difeafe, he kept aloof from his family.

I have likewife inoculated others for the Cow-pox in a neighbouring town, whilft there was in it, at the fame time, a general inoculation for the Small-pox; when not one of my patients patients felt any effects from the latter, though in a conftant communication with their infected neighbours.

Never have greater opportunities or more inftances occurred, to manifeft the certain and bleffed effects of the Cow-pox, than in this parifh and neighbourhood; for when I made my firft experiments, the Small-pox had entered the village in a dreadful form, ravaging and making devaftation in every quarter: but every one who had received the Vaccine inoculation, efcaped unhurt. Whilft a hufband has died by the former, a wife has been faved by the latter. And amongft the many hundreds that have been inoculated in the neighbourhood with the genuine Vaccine matter which I introduced, not one inftance has occurred in which it has failed of its beneficent and falutary effects.

I hope foon to hear, through the medium of your ufeful Journal, that the indefatigable and ingenious Difcoverer has felt his Country's gratitude for his labours, as they could not have been carried to their prefent perfection without infinite pains and great expence: Firft, to withftand the medical cabals that generally attend fuch difcoveries; and, fecondly, to render it fuperior to the power of Envy and Detraction to bury in obfcurity its glorious and wonderful effects.

May not only England then, but all Europe, which now receive the benefit of it, unite to exprefs, by every poffible means, their gratitude to the difcoverer of fuch a providential method of chacing from the earth one of its bittereft maladies, I am, &c.

J. S. GALE.

